# Neutrophil-to-Lymphocyte and Platelet-to-Lymphocyte Ratios in COVID-19 Patients and Control Group and Relationship with Disease Prognosis

**DOI:** 10.22088/cjim.11.0.531

**Published:** 2020

**Authors:** Mohammad Eslamijouybari, Keyvan Heydari, Iradj Maleki, Mahmood Moosazadeh, Akbar Hedayatizadeh-Omran, Lale Vahedi, Roya Ghasemian, Ali Sharifpour, Reza Alizadeh-Navaei

**Affiliations:** 1 Gastrointestinal Cancer Research Center, Non-Communicable Diseases Institute, Mazandaran University of Medical Sciences, Sari, Iran; 2 Student Research Committee, School of Medicine, Mazandaran University of Medical Sciences, Sari, Iran; 3 Gut and Liver Research Center, Non-Communicable Diseases Institute, Mazandaran University of Medical Sciences, Sari, Iran; 4 Health Sciences Research Center, Addiction Institute, Mazandaran University of Medical Sciences, Sari, Iran; 5 Department of Pathology, Faculty of Medicine, Mazandaran University of Medical Sciences, Sari, Iran; 6 Antimicrobial Resistance Research Center, Communicable Diseases Institute, Mazandaran University of Medical Sciences, Sari, Iran; 7 Department of Pulmonary and Critical Care, Mazandaran University of Medical Sciences, Sari, Iran

**Keywords:** Neutrophil-to-Lymphocyte ratio, Platelet-to-Lymphocyte ratio, COVID-19, Prognosis

## Abstract

**Background:**

The present study aimed to compare the complete blood count (CBC) indices between COVID-19 patients and the control group, and assess the relationship of these indices with COVID-19 prognosis.

**Methods:**

COVID-19 patients (confirmed by PCR or CT-Scan) who visited Imam Hospital in Sari were selected in this case-control study. The control group was selected from Tabari cohort population matched with the case group in terms of gender and age. CBC, neutrophil-to-lymphocyte ratio (NLR), platelet-to-lymphocyte ratio (PLR), and outcome of the disease (in the case group) were assessed in this study.

**Results:**

The number of participants were 527 in both case and control groups, of which, 232 (44%) were females in each arms. Platelet count, lymphocyte count, and hemoglobin concentration were also higher in the control group (P=0.000). NLR and PLR were significantly higher in COVID-19 patients compared to the control group (P=0.000). NLR had a significant relationship with the severity of the disease. NLR was two times higher in the patients who died of COVID-19 than those who recovered (P=0.000). ROC curve analysis for diagnostic values of NLR and PLR showed that the areas under the ROC curves for NLR and PLR were 0.703 (95% CI: 0.64-0.76) and 0.535 (95% CI: 0.46-06), respectively.

**Conclusion:**

NLR can be used as a prognostic marker for COVID-19 given the significant difference of NLR between those who died and recovered from COVID-19.

A large number of cases with viral pneumonia of unknown etiology have been reported since December 2019. A seafood market in Wuhan, China has been considered the likely source of this outbreak (1). The virus was found to be a member of coronavirus family on June 6, 2020 that could cause infection in humans (2, 3). The virus was named COVID-19 (2). Like SARS-CoV (the cause of severe acute respiratory syndrome) and MERS-CoV (the cause of Middle East respiratory syndrome), this newly emerged virus leads to acute respiratory distress syndrome (ARDS) (1, 4). Findings have shown that 26% of patients received ICU care and mortality was 4.3% (5). Both innate and adaptive immune systems seem to respond to viral load in the course of infection (6). B and T cells produced long-lived memory cells called immunological memory that protect hosts against secondary viral infection. Dyregulated Cytokines and chemokines are also a certain feature of disease severity as serum levels of these factors elevated in severe cases of infection (7). Careful monitoring and interpretation of these changes contribute to timely diagnosis and treatment of a variety of diseases.

CBC with differential is one of the most common test in clinical laboratories that can be measured by hematology autoanalyzers, cost-effectively, rapidly and accurately (8). Neutrophil-to-lymphocyte ratio (NLR) was found to be significantly higher in COVID-19 patients in a study. NLR > 3.13 in patients above 50 years of age is determined as a predictor of severe COVID-19 and these patients are recommended immediate admission to ICU (9).

The present study aimed to measure and compare CBC indices in the COVID-19 patients and healthy population, and assess prognostic value of these indices for the COVID-19 in an Iranian population given the newly emerged coronavirus and inadequate information on the patients infected with this virus.

## Methods

This was a cross-sectional study involving those COVID-19 patients visiting Imam Hospital of Sari in 2020. The statistical population consisted of all patients visiting Imam Hospital. The statistical sample consisted of all patients diagnosed with COVID-19 by a specialist via PCR or CT scan. The control group was selected form Tabari cohort population. Tabari cohort study profile was published previously (10). 

They were matched with the case group in terms of age and gender. The sampling techniques used to select the case and control groups were convenient and random sampling, respectively.

The study protocol was approved by the Ethics Committee of Mazandaran University of Medical Sciences (IR.MAZUMS.REC.1398.1424). Data on age, gender, NLR, and PLR were collected in the control group. In addition to the above factors, outcome of the disease was also recorded in the case group. Data analysis was performed using SPSS, chi-square, t-test, and Mann-Whitney test. ROC curve analysis was used to show diagnostic value of NLR and PLR. P<0.05 was considered significant.

## Results

The participants consisted of 527 patients with COVID-19 and 527 healthy individuals were selected from the public before the COVID-19 epidemic. Of these, 232 (44%) were males and 295 (56%) were males in both groups (P=1) given the gender-matched controls. In case group, 101 (19.2%) were under 40 years of age, 231 (43.8%) were between 41 and 60 years of age, and 195 (37%) were above 60 years of age. Similarly, 103 (19.5%) were under 40 years of age, 243 (46.1%) were between 41 and 60 years of age, and 181 (34.3%) were above 60 years of age in the control group (p=0.656). 

Table 1 shows that platelet count, lymphocyte count, and hemoglobin concentration were significantly lower in case group compared to control group. NLR and PLR were significantly higher in COVID-19 patients compared to controls. Of COVID-patients, 103 (19.5%) died of the disease. Mean NLR in the dead was significantly higher than the recovered (P=0.000). 

Mean PLR was higher in the dead compared to the recovered but the difference was not statistically significant (P=0.276) (table 2). 

**Table 1:** Distribution of CBC indices in COVID-19 patients and control group

**Variable **	**Case** **Mean±SD**	**Control** **Mean±SD**	**Pvalue**
WBC	7606.57±7841.61	6450.57±1533.87	0.001
Neutrophil	5397.96±4214.17	3791.92±1233.44	0.000
Platelet	208504.55±82330.31	247478.18±56155.26	0.000
Lymphocyte	1559.24±4630.38	2420.47±734.17	0.000
Hemoglobin	12.08±2.88	14.1±1.58	0.000

**Table 2:** Distribution of NLR and PLR in the studied individuals

	**Number**	**NLR** **Median(IQR)**	**PLR** **Median (IQR)**
Covid-19	520	3.73 (2.32-6.47)	172.88 (121.8-236.78)
Control	527	1.56 (1.21-2)	104.16 (86.07-125.75)
P-value		0.000	0.000
Covid-19 cured	422	3.45 (2.19-5.37)	169.31 (123.11-231.33)
Covid-19 died	98	6.55 (3.48-9.25)	191.19 (106.73-286.64)
P-value		0.000	0.276

**Figure 1:**
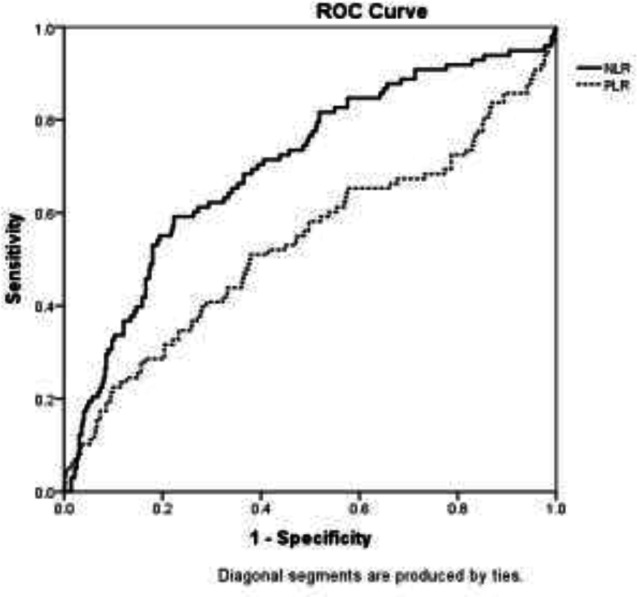
ROC curve for diagnostic value of NLR and PLR in final outcome of COVID-19 patients

ROC analysis for diagnostic value of NLR and PLR showed that the areas under the NLR and PLR curves were 0.703 (95% CI: 0.64-0.76) and 0.535 (95% CI: 0.46-06), respectively (figure 1).

## Discussion

Health professionals are searching for a suitable prognostic marker to identify those who would develop to severe cases to fairly distribute available resources among the patients since COVID-19 has spread rapidly on a global scale and no effective treatment has been discovered yet. In such a circumstance, biomarkers that can provide insight into disease progression, prognosis, and severity are of great importance. Therefore, the present study compared CBC indices between 520 patients with COVID-19 and 527 healthy individuals. 

Neutrophil counts were significantly higher and lymphocyte counts were significantly lower in the COVID-19 patients compared to the control group in the present study. Therefore, NLR was two times higher in the COVID-19 patients compared to controls. NLR was also assessed in those who died and recovered from the disease. The results showed association of NLR with disease severity and NLR seemed to be higher in severe cases of the disease. Other studies also showed increased NLR in COVID-19 patients. Assessment of NLR in critically ill patients and those with milder symptoms showed that NLR was significantly higher in critically ill patients compared to milder cases of the disease (11-13). A meta-analysis that combined data from five studies also showed a significant increase in NLR in critically ill patients (14). Similar to this study, Yang *et al.* also found out that NLR is a suitable predictive and prognostic marker for patients with COVID-19 (11). As NLR increases, severity of the disease also increases. NLR of the peripheral blood was assessed as an inflammatory marker in several studies (15, 16). A systemic inflammation suppresses T cell-mediated immunity resulting in decreased levels of T lymphocytes (17). Neutrophils are the first important leukocytes to reach the site of viral infection, enter the infected cell, and mediate tissue damage and apoptosis of virally infected cells. Neutrophils stimulate B lymphocytes to initiate humoral immunity (18). Serum levels of interleukin-6, interleukin-8, tumor necrosis factor-α, interferon gamma, and granulocyte colony stimulating factor increase following viral infection. These factors activate neutrophils leading to proliferation and migration of neutrophils to viral-infected sites (19-22). COVID-19 is a viral infection associated with systemic manifestations of pneumonia, fever, and septic shock. Gastrointestinal symptoms were less common in these patients (23).

A significant thrombocytopenia was found in COVID-19 patients in the present study. PLR was significantly higher in COVID-19 patients compared to the control group. However, PLR was not associated with the disease severity and no significant relationship was found between PLR and disease severity. Unlike this study, Qu *et al.* examined 30 patients with COVID-19 and found a significant difference in PLR between critically ill patients and patients with milder COVID-19 symptoms (24). Yang *et al.* also achieved the same results. They examined 93 patients with COVID-19 in a cross-sectional study and found a significant difference in PLR between critically ill patients and patients with milder COVID-19 symptoms (11). PLR is currently known as an inflammatory marker. Various studies have assessed the association of PLR with malignancies (25), diabetes (26), bacterial infections (27) and viral infections (28). Studies have shown that platelet counts significantly decreased in COVID-19 patients (29). PLR is calculated by dividing the platelet counts to lymphocyte counts. Therefore, both platelet and lymphocyte counts should be considered in interpretation of changes in PLR. Thrombocytopenia is a commonplace in critically ill patients, and usually suggests serious organ malfunction or physiologic decompensation as opposed to primary hematologic etiology, as well as the development of intravascular coagulopathy (30). The mechanism for thrombocytopenia is multifactorial in severe acute respiratory syndrome and refers to a combination of viral infection and mechanical ventilation leading to endothelial damage triggering platelet activation, aggregation and thrombosis in the lung, causing vast platelet consumption (31). Thrombocytopenia might be caused by platelet consumption in COVID-19 given the similarity of thrombocytopenia mechanism in COVID-19 and SARS. Coronaviruses may also directly infect bone marrow elements resulting in abnormal hematopoiesis, or trigger an auto-immune response against blood cells (31, 32). Nevertheless, significant differences between SARS and COVID-19 should not be overlooked (32, 33). In limitation, PCR was not performed for all patients due to limitations caused by COVID-19 pandemic in the present study. 

In Conclusion, given the current need for an inexpensive and accessible test that can predict prognosis of COVID-19 patients, the results of this study show high prognostic value of NLR. Given the confounding results regarding PLR, more extensive studies should be carried out to assess prognostic value of PLR. 
